# Supplementary material for: Small Molecule Anion Carriers Correct Abnormal Airway Surface Liquid Properties in Cystic Fibrosis Airway Epithelia
Source: Int J Mol Sci. 2020 Feb 21;21(4):1488. doi: 10.3390/ijms21041488 (PMC7073096; doi:10.3390/ijms21041488)
Supplement: Supplementary file 1 [file ijms-21-01488-s001.pdf]

Supplementary Information (SI)

Article

Small molecule anion carriers correct abnormal airway surface liquid properties in cystic fibrosis airway epithelia

Ambra Gianotti[1+], Valeria Capurro[1+], Livia Delpiano[1§], Marcin Mielczarek[2], María García-Valverde[2], Israel Carreira-Barral[2], Alessandra Ludovico[3], Michele Fiore[3], Debora Baroni[3], Oscar Moran[3], Roberto Quesada[2*] & Emanuela Caci[1*]

[1] UOC Genetica Medica, IRCSS Istituto Giannina Gaslini, Genova, Italy;
ambrygianotti@hotmail.com (A.G.); valeriacapurro@yahoo.it (V.C.);
livia.delpiano@gmail.com (L.D.), emanuela.caci@unige.it (E.C.)
[2] Departamento de Química, Facultad de Ciencias, Universidad de Burgos, Burgos, Spain;
marcin.mielczarek@poczta.onet.pl (M.M.); magaval@ubu.es (M.G.-V.);
icarreira@ubu.es (I.C-B.); rquesada@ubu.es (R.Q.)
[3] Istituto di Biofisica, Consiglio Nazionale Delle Ricerche (CNR), Genova, Italy;
ale.ludo89@gmail.com (A.L.); fiore@ge.ibf.cnr.it (M.F.); dbaroni@ge.ibf.cnr.it (D.B.); oscar.moran@cnr.it (O.M.)

+ These authors have equally contributed to the work.
§ Present address: Biosciences Institute, Newcastle University, Newcastle upon Tyne, United Kingdom.

1. Characterization data of compound MM34

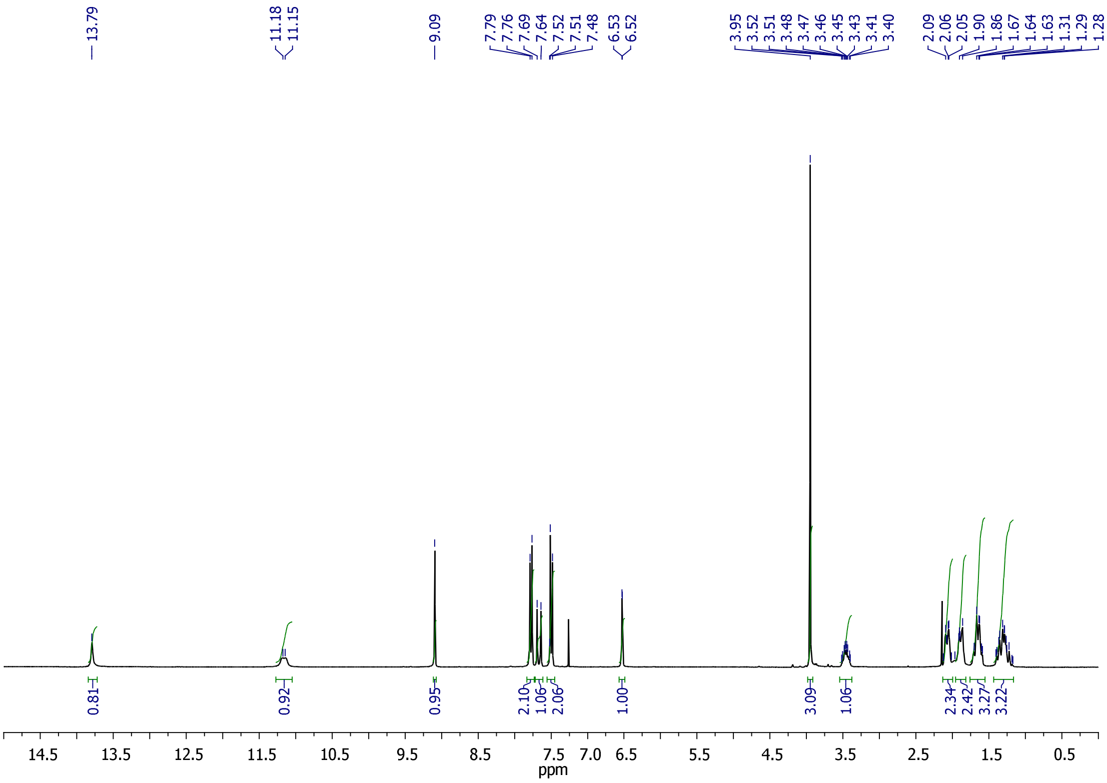

Figure S1. ^{1}H NMR spectrum (300 MHz, $CDCl_3$).

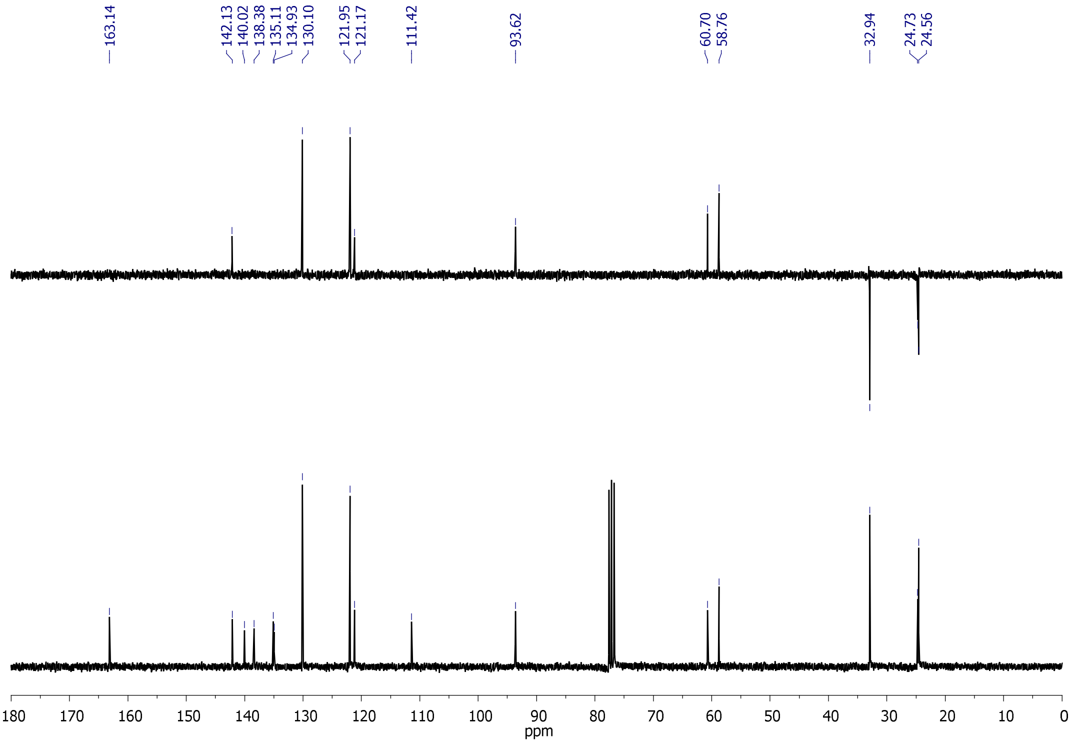

Figure S2. ^{13}C and DEPT NMR spectra (75 MHz, $CDCl_3$).

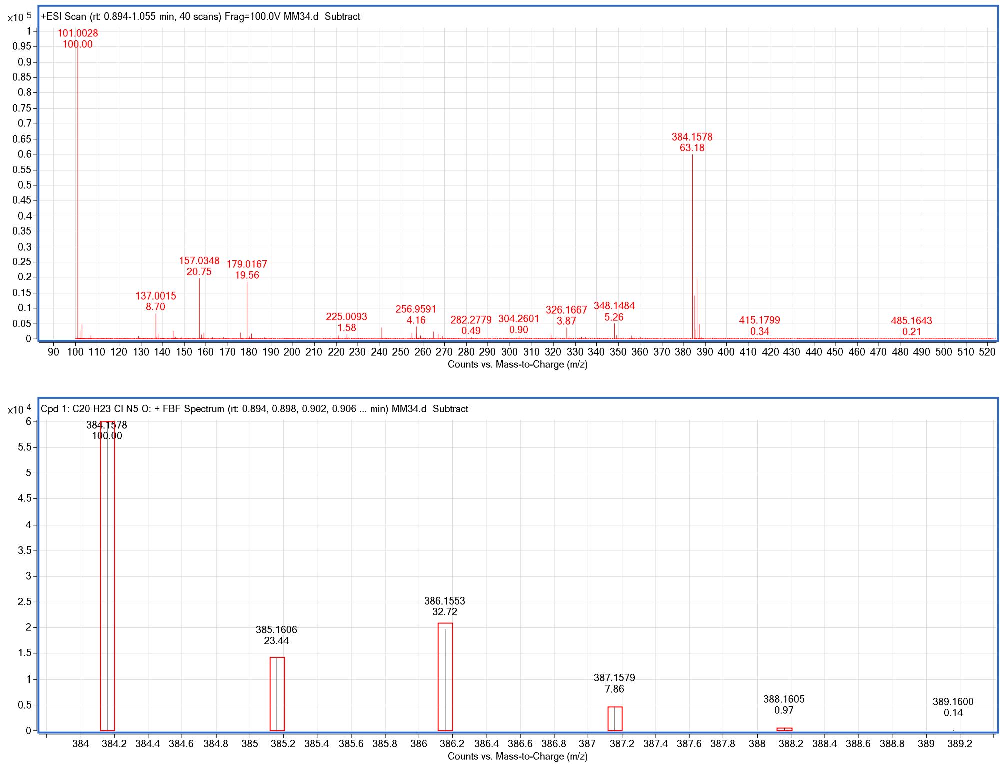

Figure S3. HR-MS (ESI$^+$) spectrum and expansion of the peak of interest.
